# Supplementary material for: A computational framework for the morpho-elastic development of molluskan shells by surface and volume growth
Source: PLoS Comput Biol. 2019 Jul 29;15(7):e1007213. doi: 10.1371/journal.pcbi.1007213 (PMC6687210; doi:10.1371/journal.pcbi.1007213)
Supplement: S1 Text — (PDF) [file pcbi.1007213.s001.pdf]

Supplementary Material - A computational framework for the morpho-elastic development of molluskan shells by surface and volume growth

Shiva Rudraraju [1], Derek E. Moulton [2], Régis Chirat [3], Alain Goriely[2], Krishna Garikipati [4*]

1 Department of Mechanical Engineering, University of Wisconsin-Madison, Madison, Wisconsin, United States of America
2 Mathematical Institute, University of Oxford, Oxford, United Kingdom
3 UMR CNRS 5276 LGL-TPE, Université Lyon1, 69622 Villeurbanne Cedex, France
4 Departments of Mechanical Engineering and Mathematics, Michigan Institute for Computational Discovery & Engineering, University of Michigan, Ann Arbor, Michigan, United States of America

* krishna@umich.edu

S1 Text. Buckling analysis of a plate

We compute here the relationship between buckling mode and active mantle width via the buckling of a plate. We consider a plate with zero reference curvature of length A in the x direction and width B in the y direction (the x and y directions here correspond to the s_2 and s_1 directions in the main text, respectively, so we are primarily interested in the case $A \gg B$). The governing equation for the transverse deformation $w(x, y)$ of the plate is [1, 2]

$$D\nabla^4 w + N w_{xx} = 0, \tag{1}$$

where D is the bending modulus and N is a compressive force due to growth in the x direction (defined as positive here). For boundary conditions, we take the plate to be clamped on one long edge, free on the other long edge, and simply supported on the two short edges. These conditions read

$$w = 0, \;\; w_{xx} + \nu w_{yy} = 0 \;\text{ on } x = 0, \; A, \tag{2}$$
$$w = 0, \;\; w_y = 0 \;\text{ on } y = 0, \tag{3}$$
$$w_{yy} + \nu w_{xx} = 0, \;\; w_{yyy} + (2 - \nu) w_{xxy} = 0 \;\text{ on } y = B, \tag{4}$$
$$\tag{5}$$

where ν is the Poisson ratio. The system (1), (2) has solution

$$w(x, y) = \sin\left(\frac{m\pi x}{A}\right) f(y)$$

where m is the buckling mode. Taking $f = \exp(i\lambda y)$ yields the characteristic equation

$$A^4\lambda^4 - \frac{A^2 m^2}{\pi^2}(\tilde{N} - 2\lambda^2) + m^4\pi^4 = 0,$$

where $\tilde{N} = N/D$. This has roots

$$\lambda_1^{\pm} = \pm i \frac{\sqrt{Am\sqrt{\tilde{N}}\pi + m^2\pi^2}}{A}, \tag{6}$$

$$\lambda_2^{\pm} = \pm \frac{\sqrt{Am\sqrt{\tilde{N}}\pi - m^2\pi^2}}{A}, \tag{7}$$

a nontrivial solution only existing if $\tilde{N} > m^2\pi^2/A^2$. The function $f(y)$ thus takes the form

$$f(y) = c_1 \exp(i\lambda_1^+ y) + c_2 \exp(i\lambda_1^- y) + c_3 \cos(\lambda_2^+ y) + c_4 \sin(\lambda_2^+ y).$$

The boundary conditions in the y-direction translate to

$$f(0) = f'(0) = 0, \quad f''(B) - \nu \frac{m^2\pi^2}{A^2} f(B) = f'''(B) - (2+\nu)\frac{m^2\pi^2}{A^2} f'(B) = 0.$$

Imposing these conditions yields an eigenvalue problem for the critical compression $\tilde{N} = \tilde{N}^*$, and the critical buckling mode is determined by finding the integer value of m at which $\tilde{N}^*$ is minimized. The points in Fig 8 of the main text were produced by fixing $A = 20$, $\nu = 0.3$, varying B between 0.5 and 2.5, and computing the critical mode at each width.

References

1. P. Howell, G. Kozyreff, and J. Ockendon. *Applied solid mechanics.* Cambridge University Press, 2009.

2. S. Timoshenko and J.M. Gere. *Theory of elastic stability* McGraw-Hill, New York, 1936.
